# A case report and literature review on primary solitary fibrous tumor of the bladder

**DOI:** 10.1097/MD.0000000000033708

**Published:** 2023-05-12

**Authors:** Tian Yu Li, Bo Zhang, Ji Zhang

**Affiliations:** a Graduate School of Dalian Medical University, Dalian, China; b Department of Medical Imaging, Taizhou People’s Hospital, Taizhou, China.

**Keywords:** bladder tumor, case report, dual-source urology CT, laparoscope, solitary fibroma tumor

## Abstract

Solitary fibrous tumors (SFT) is a rare mesenchymal tumor originating from a CD34-positive dendritic mesenchymal cell, most of which is benign, the most common sites is pleura and mediastinum, and rarely in the bladder. The clinical manifestations are mainly related to the tumor volume. When the tumor volume is large, it will compress the surrounding tissues or organs and cause corresponding symptoms. Laparoscopic Incision biopsy is effective means for diagnosing SFT.

**Patient concerns:**

A 70-year-old female patient was admitted to the hospital with a bladder neoplasm detected by computed tomography scan after experiencing intestinal obstruction 3 days following esophageal cancer surgery. She denied any history of tumor disease.

**Diagnoses:**

No abnormality was found in the physical examination and laboratory testing after admission. Ultrasound imaging showed a large solid mass with low echogenicity in the bladder. Urological computed tomography with 3D reconstruction revealed a large cystic-solid mass located on the right wall of the bladder, measuring approximately 6.8 cm × 7.1 cm × 6.5 cm, with uneven density and mild inhomogeneous enhancement after contrast administration. Cystoscopy revealed a large mucosal bulge on the right wall of the bladder and laparoscopic exploration revealed a smooth-surfaced round mass, approximately 7 cm in size.

**Interventions:**

Incision biopsy was performed to make a clear diagnosis, and appropriate tissue specimens were obtained for pathological testing.

**Outcomes:**

The patient was diagnosed as SFT according to pathology. The patient was followed up for 6 months after surgery, and no recurrence was observed.

**Lessons:**

SFT occurring in the bladder are extremely rare, and the site is scarcely reported in the relevant literature; thus, it is easy to misdiagnose and laparoscopic incision biopsy may be a good choice.

## 1. Introduction

Solitary fibrous tumors (SFT) are a rare type of tumor in the gastrointestinal stromal tumor family, first described as a distinct disease by Klemperer and Coleman.^[1]^ While SFT are most commonly found in the pleura and mediastinum, rare cases have been reported in the bladder.^[2–4]^ The diagnosis of SFT of the bladder involves a combination of clinical symptoms, imaging, and histopathological examination. The diagnosis was confirmed via immunohistochemical examination.

## 2. Case report

**Figure 1.**
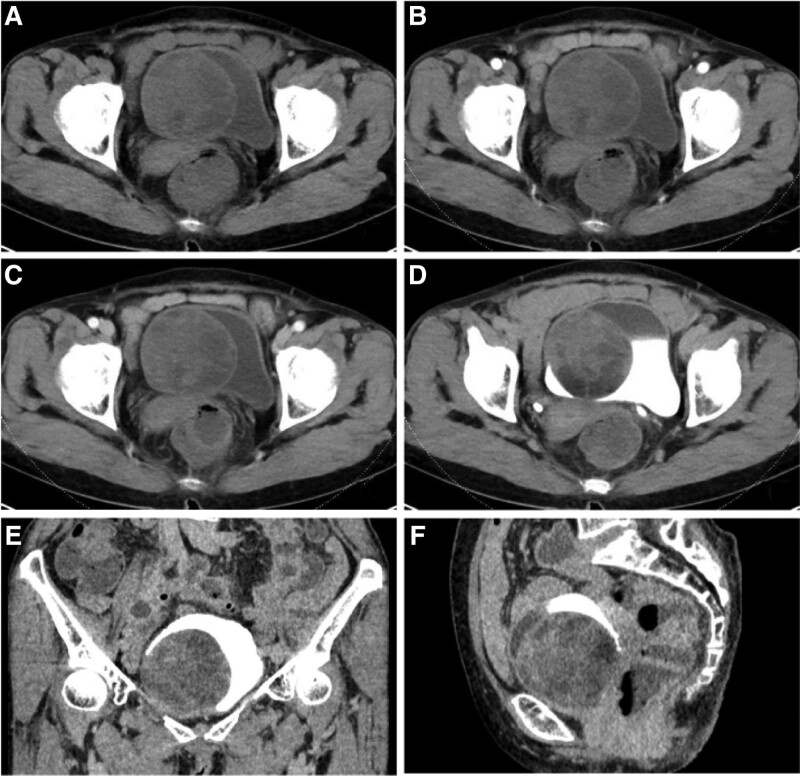
Dual-source CT urinary tract imaging. (A–F) CT contrast images of the intravesical mass. (A) Displays a round mass with smooth edges and mild-to-moderate heterogeneous enhancement in the arterial phase CT. (B and C) Demonstrate mild-to-moderate enhancement of the intravesical mass in the venous and delayed phase, with a visible patchy hypointense non-enhancing shadow. (D–F) Round hypointense filling defects in the bladder in the delayed phase on CT axial, coronal, and sagittal positions. CT = computed tomography.

**Figure 2.**
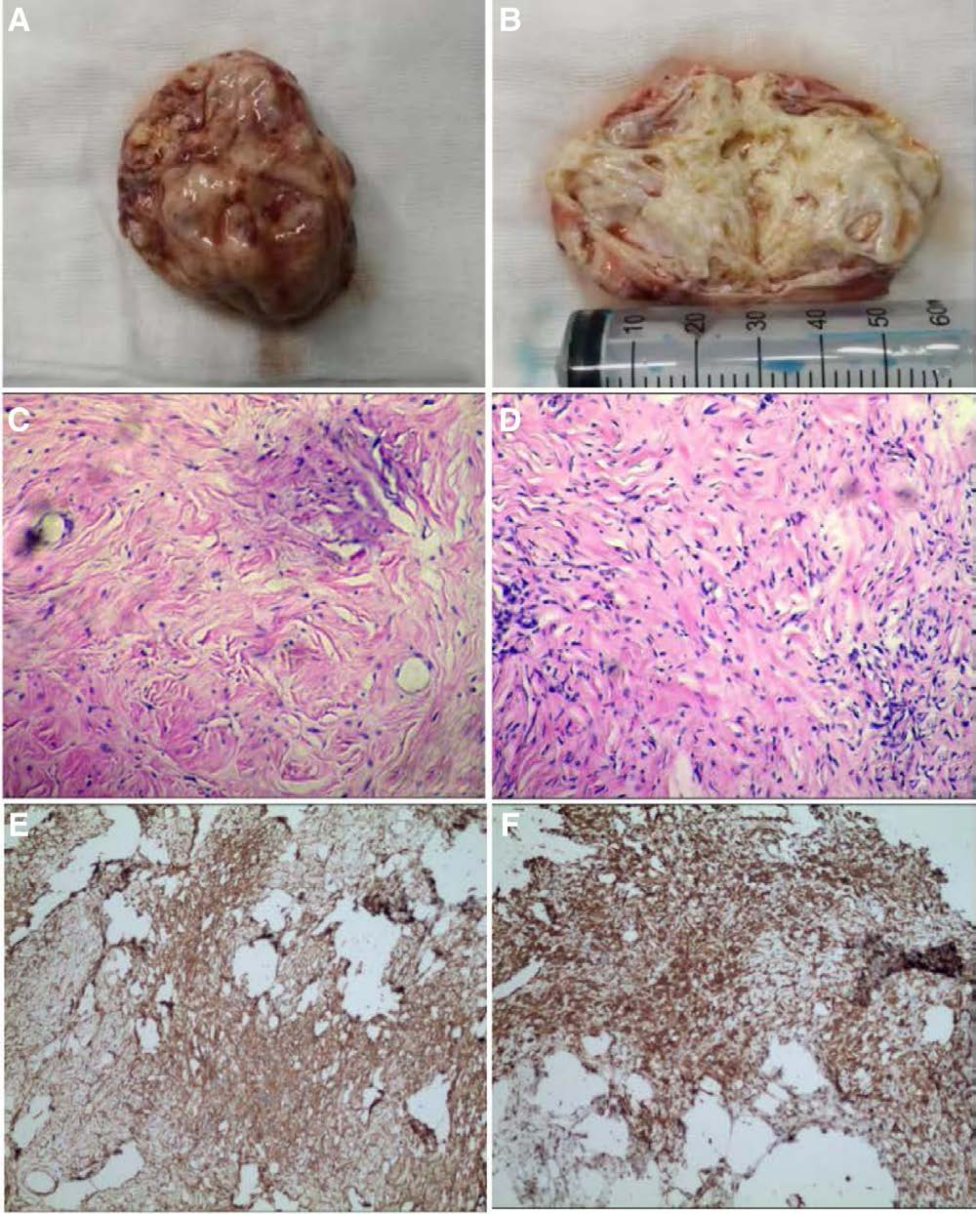
The macroscopic and microscopic views of the tumor and its immunohistochemical examination. (A and B) The tumor was grayish-white with a grayish cut surface, moderately solid, and had localized cystic cavity-like changes. (C) At low magnification, the tumor contained sparse areas of spindle-shaped tumor cells and a glassy, collagenized stroma (HE × 200). (D) At high magnification, the tumor cells were spindle-shaped with homogeneous nuclear chromatin and stained positively for nuclear markers (HE × 200). Immunohistochemical analysis showed strong, diffuse expression of CD34 in the tumor cells (SP × 200), and diffuse expression of Bcl-2 in the tumor cells with strong positivity (SP × 200).

A 70-year-old female patient was admitted to the hospital with a bladder neoplasm detected by computed tomography (CT) scan after experiencing intestinal obstruction 3 days after esophageal cancer surgery. The patient did not display any urinary symptoms such as frequency, urgency, or pain. A physical examination showed no obvious positive signs. Ultrasound imaging revealed a large solid mass with low echogenicity in the bladder and a small blood flow signal on color Doppler ultrasound. Urological CT with 3D reconstruction revealed a large cystic-solid mass located on the right wall of the bladder, measuring approximately 6.8 cm × 7.1 cm × 6.5 cm, with uneven density and mild inhomogeneous enhancement after contrast administration. The diagnosis was consistent with that of a large nerve sheath tumor (Fig. 1A–F). During cystoscopy, a large mucosal bulge was observed on the right wall of the bladder, extending from the parietal wall to near the opening of the right ureter, with a smooth surface that seamlessly merged with the rest of the bladder mucosa. Laparoscopic exploration under general anesthesia revealed a smooth-surfaced, round mass measuring approximately 7.0 cm in diameter on the right bladder wall with no adhesions to surrounding tissues. After the central longitudinal incision, the mass was cystic and located solely within the muscular layer, without extending into the mucosal layer. The mass was completely excised and sent for pathological examination. Gross specimen analysis showed a grayish-white mass measuring approximately 6.5 cm × 6  cm× 4 cm (Fig. 2A and B). The mass was solid with localized cystic changes measuring up to 1.5 cm in diameter upon longitudinal dissection. Microscopic examination revealed a well-defined tumor with alternating areas of high and low cell density, as well as hemangioepithelioma-like areas (Fig. 2C). At a high magnification, spindle-shaped tumor cells with limited cytoplasm and reddish staining were observed. The nuclei of the cells were uniform in chromatin, with inconspicuous nucleoli, and no signs of nuclear fission were observed (Fig. 2D). Immunohistochemistry revealed that the tumor cells were positive for vimentin (+++) and CKpan (+), and negative for SMA (−), CD34 (+++) (Fig. 2E), and BCL-2 (++) (Fig. 2F). CD99 was positive (++), whereas EMA, Desmin, and S100 were negative. Stata-6 was strongly positive (+++) and Ki-67 showed a 1% positive index. Based on the HE morphology and immune-enzymatic labeling results, the diagnosis was solitary fibrous tumor. The patient was followed-up for 6 months after surgery, and no recurrence was observed.

## 3. Discussion

SFT were initially thought to arise from the mesothelial or submesothelial mesenchymal cells in the pleura. However, subsequent studies using immunohistochemical techniques and electron microscopy have shown that SFT originates from dendritic mesenchymal cells that are CD34-positive and capable of differentiating into fibroblasts, myofibroblasts, and vascular epithelial cells.^[5–7]^ Although SFT can occur in various locations in the body, primary bladder SFT is rare, and patients may present with urinary symptoms and pelvic compression or be asymptomatic. In this case, the diagnosis was incidental during the evaluation of other diseases, and the patient did not have significant urinary symptoms.

Ultrasound of the SFT in the bladder typically shows an echogenic mass with low or heterogeneous echogenicity with well-defined borders. On CT scan, a large solid soft tissue mass with mixed density was observed. On contrast CT scan, nodular or map-like heterogeneous enhancement in the arterial phase, with cystic, hemorrhagic, or necrotic areas can be seen. The enhancement of the mass gradually increase in the venous and delayed phases and the density of the lesion became more homogeneous with a “fast-in, slow-out” pattern.^[8]^ In this case, CT showed a homogeneous cystic solid mass with no significant enhancement of the solid component in the arterial phase, slight enhancement in the venous phase, and gradual enhancement in the delayed phase. These findings were not consistent with the literature and may be related to the presence of more mucinous-like changes in tumors with fewer tumor cells. The SFTs usually appear as solitary lesions with clear borders on MRI with low signal intensity on T1 WI and mixed signal intensity on T2 WI. Some researchers believe that the low signal intensity area on T1 WI is due to the collagen-rich fibrous matrix and sparse cellular structure in the lesion, while the area with iso-intensity is composed of the cell-rich area.^[9]^ The contrast scan shows that the SFT has obvious inhomogeneous, sustained, and delayed enhancement. The sustained enhancement area corresponds to histologically cell-rich and vascular-rich areas, and the delayed enhancement area corresponds to areas with sparse collagen tissue or cellular structure.^[10,11]^ However, MRI was not performed in this patient prior to surgery and was not included in the clinical data.

The gross specimen of bladder SFT is usually a well-defined, solitary solid mass with a round, oval, or slightly irregular shape. The tumor section was grayish to tan in color, with visible hemorrhages and cystic changes. The microscopic morphology of SFT is characterized by the presence of a mucus-like interstitial matrix with alternating sparse and abundant cellular areas, coarse collagen fibers, and a branching hemangioepithelioma-like vascular pattern. The cells were spindle-shaped or round, with no significant atypia. Mitotic are generally, mitotic cells do not exceed 3 nuclei per 10 high-power fields. Immunohistochemical analysis revealed positivity for CD34 and Bcl-2, which are the most specific and accurate markers,^[12]^ but not for Desmin, CK, or S-100. In this case, the pathological presentation was consistent with typical SFT.

SFT of the bladder must be differentiated from several other lesions, including: ① Bladder cancer, a common type of malignancy among middle-aged and elderly men. It is typically located in the bladder triangle and appears as a soft tissue mass with a cauliflower-like growth pattern or limited cavity thickening. Bladder cancer is prone to cystic necrosis and shows significant inhomogeneous enhancement on CT scans with or without lymph node metastasis. It can be easily differentiated from SFT of the bladder. ② Paraganglioma of the bladder, a tumor with a rich blood supply, which appears on imaging as a solid mass in the bladder wall that protrudes into the lumen with a clear border and wide base. It is common for paragangliomas to have cystic and necrotic changes, and the enhancement of the lesion is obvious, with characteristic abnormal capsule enhancement. This type of tumor is often associated with paroxysmal hypertension, particularly a transient increase in blood pressure after urination.^[13,14]^ ③ Neurofibroma or nerve sheath tumor of the bladder: appears as a round mass with smooth margins in the bladder. The nature is either cystically or cystically solid. On CT, it appears equal or slightly hypodensity with progressive enhancement and gradual expansion. Scattered dotted or pinpoint enhancing vessels can be observed inside the lesion.^[15]^ It can be difficult to differentiate from mild SFT, but it is different from the typical bladder SFT. ④ Metastases may present as polyp-like or nodular lesions and, can be easily differentiated from bladder SFT based on medical history and clinical symptoms. Additionally, a specific fusion gene, NAB2-STAT6, has been found in both benign and malignant SFTs, which can be used to differentiate them from most spindle cell soft tissue tumors.^[16,17]^

The primary treatment for bladder SFT is complete surgical resection, with consideration for repeat resection in cases of local recurrence. The most important factor affecting prognosis is whether the tumor has been completely removed. Most SFT cases have a good prognosis with no recurrence or metastasis after surgery. However, a few histologically benign SFTs may become malignant upon recurrence,^[18]^ making the prognosis difficult to predict. The size of the tumor may not determine the prognosis, but rather the severity of the patient’s symptoms. The simple histological pattern may not be sufficient to determine its prognosis.^[19]^ Long-term follow-up is necessary for these patients. SFT may not respond well to radiation or chemotherapy, but targeted therapy, such as imatinib, has shown promising results in some studies.^[20]^

In conclusion, the diagnosis of bladder SFT relies on clinical symptoms, imaging features, and pathological examinations. The confirmation of the diagnosis is typically achieved through immunohistochemical testing, using CD34 and Bcl-2 as the most specific and sensitive immunomarkers. Bladder SFTs are generally biologically benign, and complete surgical resection is the primary treatment option. However, a small proportion of patients may exhibit local recurrence or malignant transformation, emphasizing the importance of long-term follow-up after surgery.

## Acknowledgments

We would like to thank all the staff and nurses for their kind cooperation. We would also like to thank the patient.

## Author contributions

**Supervision:** Tian Yu Li, Bo Zhang, Ji Zhang.

Writing – original draft: Tianyu Li.

Writing – review & editing: Tianyu Li, Bo Zhang, Ji Zhang.
